# Impact of the New Generation Reconstituted Surfactant CHF5633 on Human CD4^+^ Lymphocytes

**DOI:** 10.1371/journal.pone.0153578

**Published:** 2016-04-14

**Authors:** Markus Fehrholz, Kirsten Glaser, Silvia Seidenspinner, Barbara Ottensmeier, Tore Curstedt, Christian P. Speer, Steffen Kunzmann

**Affiliations:** 1 University Children’s Hospital, University of Wuerzburg, Wuerzburg, Germany; 2 Department of Molecular Medicine and Surgery, Karolinska Institutet at Karolinska University Hospital, Stockholm, Sweden; Centre Hospitalier Universitaire Vaudois, FRANCE

## Abstract

**Background:**

Natural surfactant preparations, commonly isolated from porcine or bovine lungs, are used to treat respiratory distress syndrome in preterm infants. Besides biophysical effectiveness, several studies have documented additional immunomodulatory properties. Within the near future, synthetic surfactant preparations may be a promising alternative. CHF5633 is a new generation reconstituted synthetic surfactant preparation with defined composition, containing dipalmitoyl-phosphatidylcholine, palmitoyl-oleoyl-phosphatidylglycerol and synthetic analogs of surfactant protein (SP-) B and SP-C. While its biophysical effectiveness has been demonstrated *in vitro* and *in vivo*, possible immunomodulatory abilities are currently unknown.

**Aim:**

The aim of the current study was to define a potential impact of CHF5633 and its single components on pro- and anti-inflammatory cytokine responses in human CD4^+^ lymphocytes.

**Methods:**

Purified human CD4^+^ T cells were activated using anti CD3/CD28 antibodies and exposed to CHF5633, its components, or to the well-known animal-derived surfactant Poractant alfa (Curosurf^®^). Proliferative response and cell viability were assessed using flow cytometry and a methylthiazolyldiphenyltetrazolium bromide colorimetric assay. The mRNA expression of IFNγ, IL-2, IL-17A, IL-22, IL-4, and IL-10 was measured by quantitative PCR, while intracellular protein expression was assessed by means of flow cytometry.

**Results:**

Neither CHF5633 nor any of its phospholipid components with or without SP-B or SP-C analogs had any influence on proliferative ability and viability of CD4^+^ lymphocytes under the given conditions. IFNγ, IL-2, IL-17A, IL-22, IL-4, and IL-10 mRNA as well as IFNγ, IL-2, IL-4 and IL-10 protein levels were unaffected in both non-activated and activated CD4^+^ lymphocytes after exposure to CHF5633 or its constituents compared to non-exposed controls. However, in comparison to Curosurf^®^, expression levels of anti-inflammatory IL-4 and IL-10 mRNA were significantly increased in CHF5633 exposed CD4^+^ lymphocytes.

**Conclusion:**

For the first time, the immunomodulatory capacity of CHF5633 on CD4^+^ lymphocytes was evaluated. CHF5633 did not show any cytotoxicity on CD4^+^ cells. Moreover, our *in vitro* data indicate that CHF5633 does not exert unintended pro-inflammatory effects on non-activated and activated CD4^+^ T cells. As far as anti-inflammatory cytokines are concerned, it might lack an overall reductive ability in comparison to animal-derived surfactants, potentially leaving pro- and anti-inflammatory cytokine response in balance.

## Introduction

Airway instillation of exogenous surfactant preparations has drastically reduced mortality and morbidity of preterm infants suffering from respiratory distress syndrome (RDS) due to a deficiency of pulmonary surfactant [1, 2]. Pulmonary surfactant ensures proper gas exchange in alveoli of mammalian lungs by reducing surface tension of the alveolar epithelium [3]. It is a complex mixture of 90% lipids and about 10% surfactant-specific proteins, namely surfactant protein (SP)-A, -B, -C, and -D [4, 5]. Natural surfactant preparations are derived from porcine or bovine lungs using organic solvents, thus maintaining residual hydrophobic SP-B and SP-C but lacking hydrophilic SP-A and SP-D [4]. SP-B and SP-C have been proven to significantly improve the spreading of the exogenously applied surfactant inside the lungs, thereby constituting survival benefit over protein-free preparations [6].

An increased understanding of the molecular mechanisms involved in the formation of the alveolar surfactant layer led to the development of synthetic surfactant preparations with defined compositions [7]. CHF5633 is a new generation reconstituted synthetic surfactant containing a simple, 1:1 mixture of dipalmitoyl-phosphatidylcholine (DPPC), the major constituent of pulmonary surfactant [8], and palmitoyl-oleoyl-phosphatidylglycerol (POPG) in combination with additional synthetic peptide analogs to SP-B (0.2%) and SP-C (1.5%). The SP-B analog consists of 34-amino acids derived from the two parts (8–25 and 63–78) of the full-length natural SP-B but with the methionines substituted with leucines. The SP-C analog is a 33-amino acid peptide similar to native SP-C but with an N-terminal truncation, palmitoylcysteines substituted with serines, valines and the methionine in the hydrophobic C-terminal helical segment with leucines and the leucine in position 12 with a lysine [9]. CHF5633 has recently been shown to be equally effective in treating extremely immature newborn lambs in comparison to other standard surfactant preparations [9] and in treating experimentally induced meconium aspiration syndrome in newborn pigs [10]. Moreover, it revealed superior resistance to inactivation in preterm lambs in comparison to Curosurf^®^ [11] and has currently been subject to a first clinical trial [12].

Besides improving lung function and oxygenation, various surfactant preparations have been shown to modulate innate and adaptive immune responses, thereby potentially influencing lung inflammatory processes [4]. It was shown that surfactant preparations are able to decrease pro-inflammatory cytokine and chemokine release, oxidative burst activity, and nitric oxide production in lung inflammatory cells such as alveolar monocytes and macrophages, and may also affect lymphocyte proliferative response [4]. Notably, most of those studies focused on monocytes or macrophages with tumor necrosis factor-α (TNF-α) being the primary target [13–21]. The potential impact of surfactant preparations on lymphocytic cytokine responses, especially those on CD4^+^ T helper (Th) cells, has been less examined, so far. Studies available analyzed potential effects of surfactant preparations on proliferation [22–34] and cell viability [22, 26, 27, 32] of peripheral blood mononuclear cells (PBMCs), but did not focus on purified lymphocytes alone.

CD4^+^ T cells and their subsets represent an important part of the adaptive immunity in the lung and mediate their effector function via several secreted cytokines which in turn modulate fate and function of other cells including T cells, as well as B cells, dendritic cells, macrophages, epithelial cells, and fibroblasts [35]. The four major CD4^+^ T cell subsets having been identified so far, are Th1 cells, expressing IFNγ and IL-2, Th2 cells, characterized by the expression of IL-4 and IL-13, Th17 cells expressing IL-17A and IL-22, and T-regulatory cells, which are known to produce IL-10 [36]. IL-2, IFNγ, and IL17A are generally considered pro-inflammatory cytokines. IL-2 has the ability to function as a growth factor for T cells as well as natural killer cells [37], whereas IFNγ is able to activate macrophages and induce other important pro-inflammatory parameters such as TNF-α [38]. IL17A is known to induce many inflammatory cytokines and chemokines in myeloid and mesenchymal cells [39]. In contrast, IL-4 and IL-10 are deemed to be anti-inflammatory cytokines suppressing the pro-inflammatory cytokine milieu through their corresponding receptors which are widely expressed among various cell types including monocytes and macrophages [40–42]. As far as IL-22 is concerned, both pro- as well as anti-inflammatory properties have been discussed [43, 44].

With regard to CHF5633, data on potential effects on lymphocytic effector functions are missing, so far. A beneficial impact of surfactant preparations on pro- and anti-inflammatory cytokine-release of CD4^+^ lymphocytes present in the lung would be desirable side effects. The aim of the current study was to characterize pro- and anti-inflammatory cytokine responses of human CD4^+^ T cells following exposure to the new reconstituted synthetic surfactant preparation CHF5633 and to the known, animal derived surfactant preparation Curosurf^®^.

## Materials and Methods

### Antibodies

Antibodies to CD4 (clone OKT4, APC-conjugated), IL-2 (clone MQ1-17H12, PE-Cy7-conjugated), IL-4 (clone MP4-25D2, PE-conjugated), and IL-10 (clone JES3-9D7, Alexa Fluor 488-conjugated) were all purchased from BioLegend (San Diego, CA). The antibody to IFNγ (clone 4S.B3, PerCP-Cy5-5-conjugated) was purchased from BD Biosciences (Franklin Lakes, NJ).

### Surfactant preparations and components

Curosurf^®^, CHF5633 and its components DPPC and POPG, as well as 1:1 mixtures of DPPC and POPG with or without peptide analogs to SP-B (0.2%) and SP-C (1.5%) were supplied by Chiesi Farmaceutici S.p.A. (Parma, Italy).

### Isolation and cultivation of CD4^+^ T cells

PBMCs were accumulated from randomized leukocyte concentrates obtained from apheresis products of anonymized healthy adult donors from the university hospital’s Department of Immunohematology and Transfusion Medicine, University of Wuerzburg, as described previously [45]. This study has been approved by the Ethic Committee of the Medical Faculty of Wuerzburg. Leukocytes were isolated using Ficoll-Paque (LINARIS Biologische Produkte GmbH, Dossenheim, Germany) for 25 min at 530×g. CD4^+^ lymphocytes were further enriched by positive isolation using the Dynabeads^®^ CD4 Positive Isolation Kit (Life Technologies, Carlsbad, CA) according to the manufacturers’ instructions. The purity of the isolated CD4^+^ lymphocytes was > 97%. CD4^+^ cells were maintained in RPMI 1694 and DMEM (Sigma-Aldrich), respectively, supplemented with 10% fetal bovine serum, 100 U/mL penicillin and 100 μg/mL streptomycin (Gibco, Life Technologies, Carlsbad, CA). Incubation was carried out at 37°C in a humidified atmosphere with 5% CO_2_.

### Activation and treatment of CD4^+^ T cells

CD4^+^ lymphocytes were activated by seeding 2×10^5^ cells per well in 96 well plates (Greiner) which were previously coated for 72 h at 4°C with anti CD3 (1 μg/mL; clone MEM-57; Abcam, Cambridge, United Kingdom) and anti CD28 (1 μg/mL; clone CD28.2; Abcam) antibodies. In addition, cells were either left untreated or incubated with 100 μg/mL Curosurf^®^, 100 μg/mL CHF5633, or different combinations of its single components in concentrations of 100 μg/mL for the respective time as indicated. Only POPG was applied using 50 μg/mL to ensure proportionate concentrations present in 100 μg/mL CHF5633.

### RNA extraction and reverse-transcription PCR (RT-PCR)

For RNA extraction, 6 wells of activated CD4^+^ T cells were treated as indicated, pooled and sedimented by centrifugation at 210×g for 5 min. Cells were washed with Dulbecco’s Phosphate Buffered Saline (DPBS; Sigma-Aldrich) and total RNA was isolated using NucleoSpin^®^ RNA II Kit (Macherey-Nagel, Dueren, Germany) according to the manufacturer’s protocol. For quantification of total RNA, a Qubit^®^ 2.0 Fluorometer (Invitrogen, Life Technologies) was used. Total RNA was eluted in 60 μL nuclease-free H2O and stored at -80°C until reverse transcription. For RT-PCR, 0.2 to 0.6 μg of total RNA was reverse transcribed using High Capacity cDNA Reverse Transcription Kit (Applied Biosystems, Life Technologies, Carlsbad, CA) according to the manufacturer’s instructions. First strand cDNA was stored at -20°C upon analysis.

### Quantitative PCR (qPCR)

For quantitative detection of cytokine and B2M mRNA, first strand cDNA was diluted 1 to 10 with deionized, nuclease-free H_2_O (Sigma) and 10 μL were analyzed in duplicates of 25 μL reactions using 12.5 μL iTaq^™^ Universal SYBR^®^ Green Supermix (Bio-Rad Laboratories, Hercules, CA), 0.5 μL deionized H_2_O, and 1 μL of a 10 μM solution of forward and reverse primers as indicated in Table 1. PCRs were performed on an Applied Biosystems^®^ 7500 Real-Time PCR System (Life Technologies) using a 2-step PCR protocol after an initial denaturation at 95°C for 10 min with 40 cycles of 95°C for 15 s and 60°C for 1 min. A melt curve analysis was performed at the end of every run to verify single PCR products. Levels of cytokine mRNAs were normalized to those of β-2-microglobulin (B2M), which was pre-evaluated among five frequently used housekeeping genes (peptidylprolyl isomerase (PPI) A, PPIB, B2M, ribosomal protein L13a (RPL13A), hypoxanthine phosphoribosyltransferase 1 (HPRT1), and cancer susceptibility candidate 3 (CASC3)) for analysis in CD3/CD28 activated human CD4^+^ lymphocytes. Mean fold changes in mRNA expression were calculated by the ΔΔC_T_ method by Livak and Schmittgen [46].

**Table 1:** Primers for qPCR.

Gene symbol	Sequence accession #	Orientation	Sequence [5’ to 3’]	Amplicon length [bp]
B2M	NM_004048	forward	CCAGCAGAGAATGGAAAGTC	269
		reverse	GATGCTGCTTACATGTCTCG	
IFNγ	NM_000619	forward	GTTCTCTTGGCTGTTACTG	144
		reverse	CTGTCACTCTCCTCTTTCC	
IL-2	NM_000586	forward	TCTGGAACTAAAGGGATCTG	115
		reverse	GTGTTGAGATGATGCTTTGAC	
IL-4	NM_000589	forward	AACTGAGAAGGAAACCTTCTG	182
		reverse	GGACAGGAATTCAAGCCC	
IL-10	NM_000572	forward	GCTGTCATCGATTTCTTCC	112
		reverse	GTCAAACTCACTCATGGCT	
IL-17A	NM_002190	forward	ATTGGTGTCACTGCTACTG	140
		reverse	CGGTTATGGATGTTCAGGT	
IL-22	NM_020525	forward	CTATATCACCAACCGCACCT	111
		reverse	CTCACTCATACTGACTCCGT	

### Cell vitality assay

To measure cell viability after treatment with surfactant preparations, Methylthiazolyldiphenyltetrazolium bromide (MTT) was used. For this purpose, 2 wells of activated CD4^+^ T cells were treated for the appropriate time as indicated, pooled and the same volume of cell culture medium containing additional 2.4 mM MTT was applied to the cells. After incubation at 37°C for 30 min cells were sedimented by centrifugation at 210×g for 5 min to remove the MTT-medium and then 200 μL isopropanol was added to the cell pellet to dissolve purple formazan by vortexing. Optical density was measured in triplicates in 96 well plates (Greiner) with an MR 5000 microplate reader (Dynatech, Santa Monica, CA) at 550 nm. Untreated cells were considered 100% vital and used as reference.

### Proliferation assay

The amount of proliferated CD4^+^ lymphocytes after stimulation with anti CD3/CD28 antibodies was measured using carboxyfluorescein succinimidyl ester (CFSE). Prior to activation and exposure to surfactant, CD4^+^ T cells were treated with 5 μM of CellTrace^™^ CSFE (Life Technologies) according to manufacturer’s protocol. The amount of proliferated cells was assessed via flow cytometry after 72 h treatment by using unstimulated cells as a negative control.

### Flow cytometry

To accumulate *de novo* synthesized cytokines in the Golgi apparatus, 10 μg/mL Brefeldin A (Sigma-Aldrich) was added to the cells 16 h prior to the end of the indicated incubation time. Subsequently, CD4^+^ lymphocytes were washed with PBS containing 1% human serum (HS), stained with anti CD4 antibody for 30 min at 4°C, washed twice with PBS, and stained with fixable viability dye (eFluor^®^ 780; eBioScience) for 30 min in the dark at 4°C. Cells were then washed again with PBS 1% HS and fixed for 30 min using fixation buffer (BioLegend). For an optimized permeabilization after treatment with surfactant, CD4^+^ lymphocytes were resuspended in ice-cold methanol as previously described [45]. After incubation for 30 min on ice in the dark, cells were washed twice with PBS 1% HS followed by staining with directly conjugated anti-cytokine antibodies in PBS 1% HS for 45 min at RT in the dark. After a final wash cells were resuspended in PBS 1% HS for flow cytometry using a FACSCanto^™^ II flow cytometer (BD Biosciences, Franklin Lakes, NJ). Instrument set-up and compensation/calibration were performed prior to data acquisition. Results for cytokine positive cells (mean ± SD) are expressed as the percentage of the respective subpopulation. A minimum of 50,000 lymphocyte-gated events was recorded and analyzed with FACSDiva v6.1.3 software (BD Biosciences). Events were gated on lymphocytes via forward and side scatter and for CD4^+^ Viability-dye^-^ cells after exclusion of doublets. Results are presented as percentage CD4^+^ lymphocytes positive for IFNγ, IL-2, IL-4, and IL-10 respectively.

### Statistical analysis

Results are given as means ± SD. Unless otherwise stated, data were analyzed using Kruskal-Wallis test with Dunn’s multiple comparison post hoc test. A *p*-value ≤ 0.05 was considered significant. All statistical analyses were performed using Prism^®^ version 6 (GraphPad Software, San Diego, CA).

## Results

### Effect on vitality of activated CD4^+^ lymphocytes

**Fig 1:**
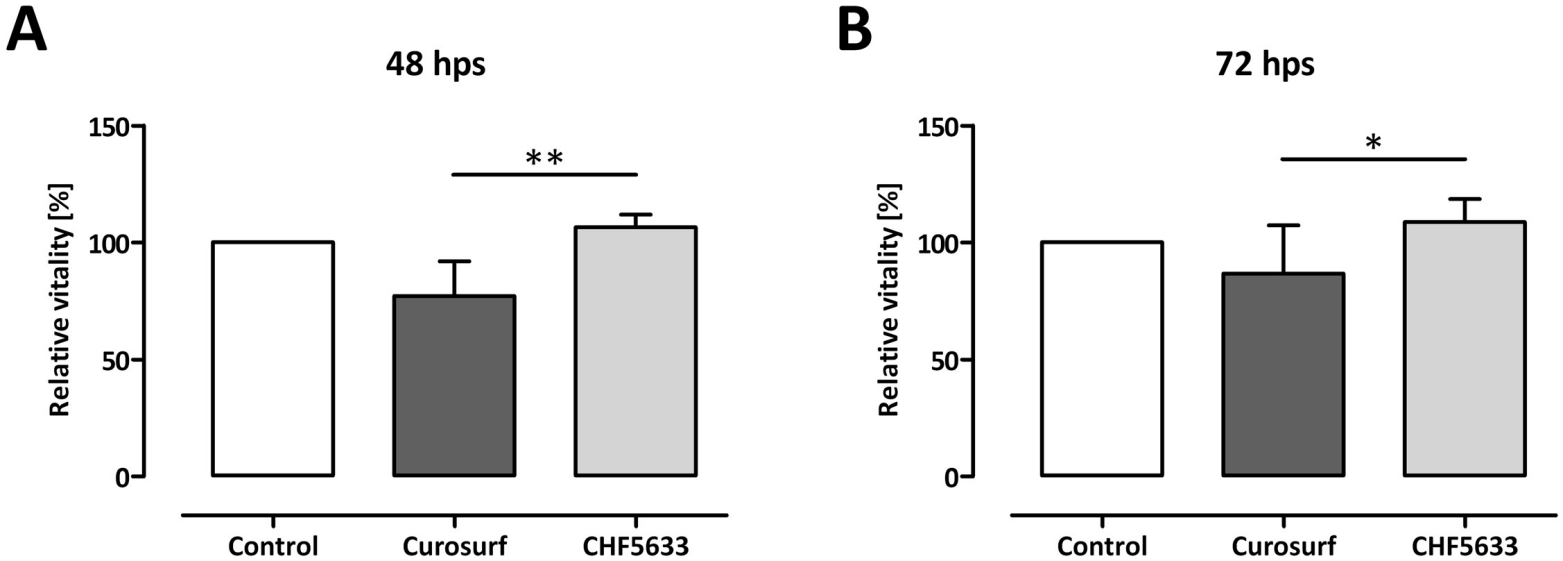
Influence of surfactant preparations on vitality of activated CD4^+^ lymphocytes. CD3/CD28 activated CD4^+^ lymphocytes were either left untreated or incubated with 100 μg/mL Curosurf^®^ or CHF5633 for 48 h (**A**) or 72 h (**B**), followed by measurement of cell viability by using MTT. Means + SD of n = 5 independent experiments are shown. Hps, hours post stimulation; * *p* < 0.05; ** *p* < 0.01.

We assessed vitality of CD4^+^ lymphocytes following a 48 h (Fig 1A) and 72 h (Fig 1B) exposure to CHF5633 and Curosurf^®^. Neither CHF5633 nor Curosurf^®^ led to significant modifications of cell viability in comparison to untreated controls at both time points. Single components of CHF5633 or different mixtures thereof also had no influence on vitality in comparison to untreated cells or cells treated with CHF5633 alone (S1 Fig). A significant and slightly higher cell viability was measured for CHF5633 if directly compared to Curosurf^®^ (Fig 1).

### Influence on proliferation of activated CD4^+^ lymphocytes

**Fig 2:**
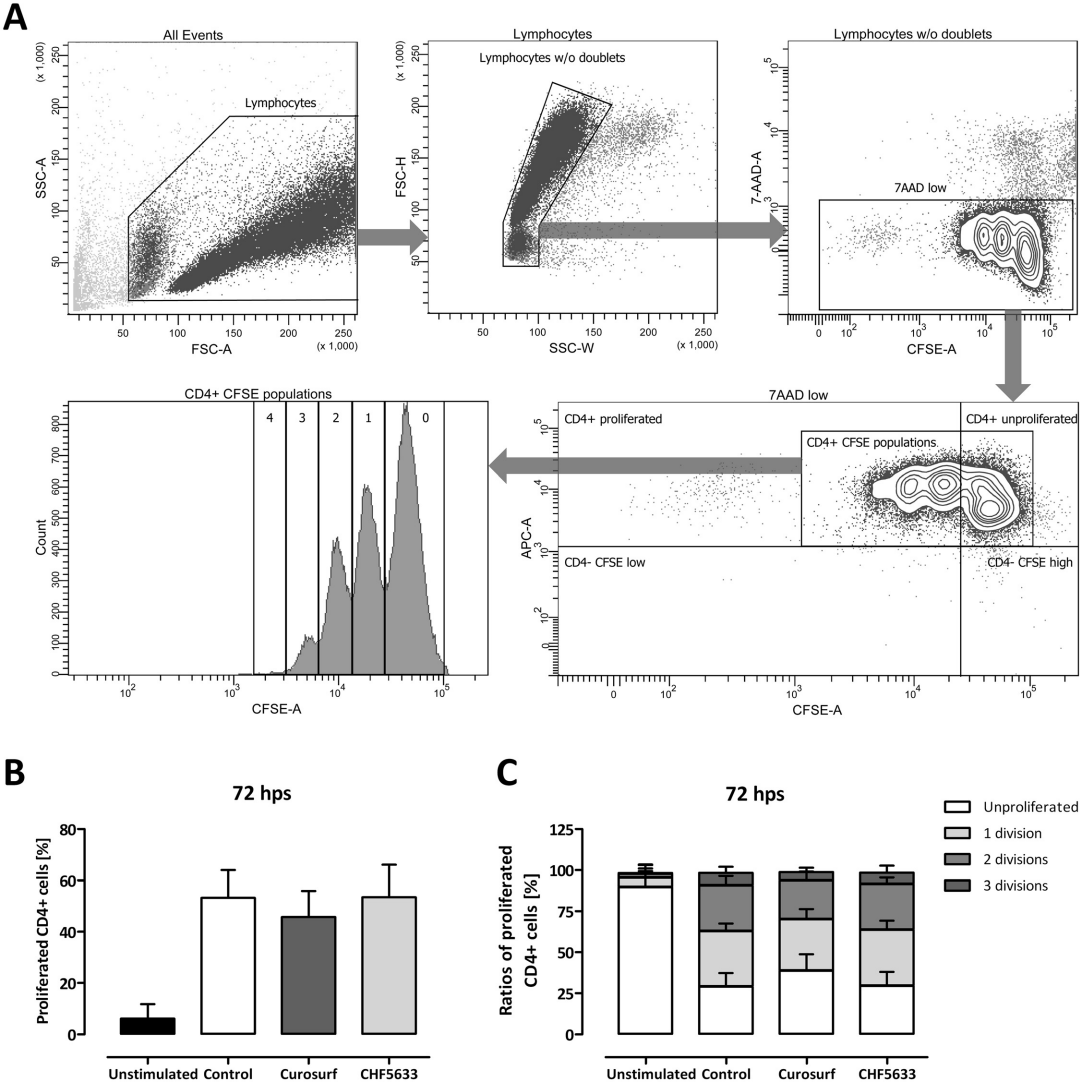
Influence of surfactant preparations on proliferation ability of activated CD4^+^ lymphocytes. CD3/CD28 activated CD4^+^ lymphocytes were either left untreated or incubated with 100 μg/mL Curosurf^®^ or CHF5633 and 72 h later proliferation was determined by means of CFSE. Vital CFSE-positive CD4^+^ lymphocytes were gated as shown in **A**. Numbers of regions spanning CFSE peaks in **A** indicate passed divisions. No significant differences between total proliferated cells (**B**) or ratios of different amounts of divisions (**C**) were found after treatment with Curosurf^®^ or CHF5633 in comparison to untreated cells. Means + SD of n = 6 independent experiments are shown. Hps, hours post stimulation.

CFSE-stained non-exposed and CHF5633- or Curosurf^®^ exposed, activated CD4^+^ lymphocytes were analyzed by means of flow cytometry to estimate the amount of divisions after a 72 h treatment. The gating strategy for these experiments is shown in Fig 2A. We could not detect any influence of either Curosurf^®^ or CHF5633 on total amount of proliferated cells (Fig 2B), or on the amount of passed divisions after activation (Fig 2C). For every performed experiment, the amount of cells reaching 4 divisions was below 1% and therefore not included in the analysis. Single components of CHF5633 or different mixtures thereof also had no influence on either the amount of proliferated cells or the passed divisions in comparison to untreated cells or cells treated with CHF5633 alone (S2 Fig).

### Cytokine mRNA expression in resting and activated CD4^+^ lymphocytes

**Fig 3:**
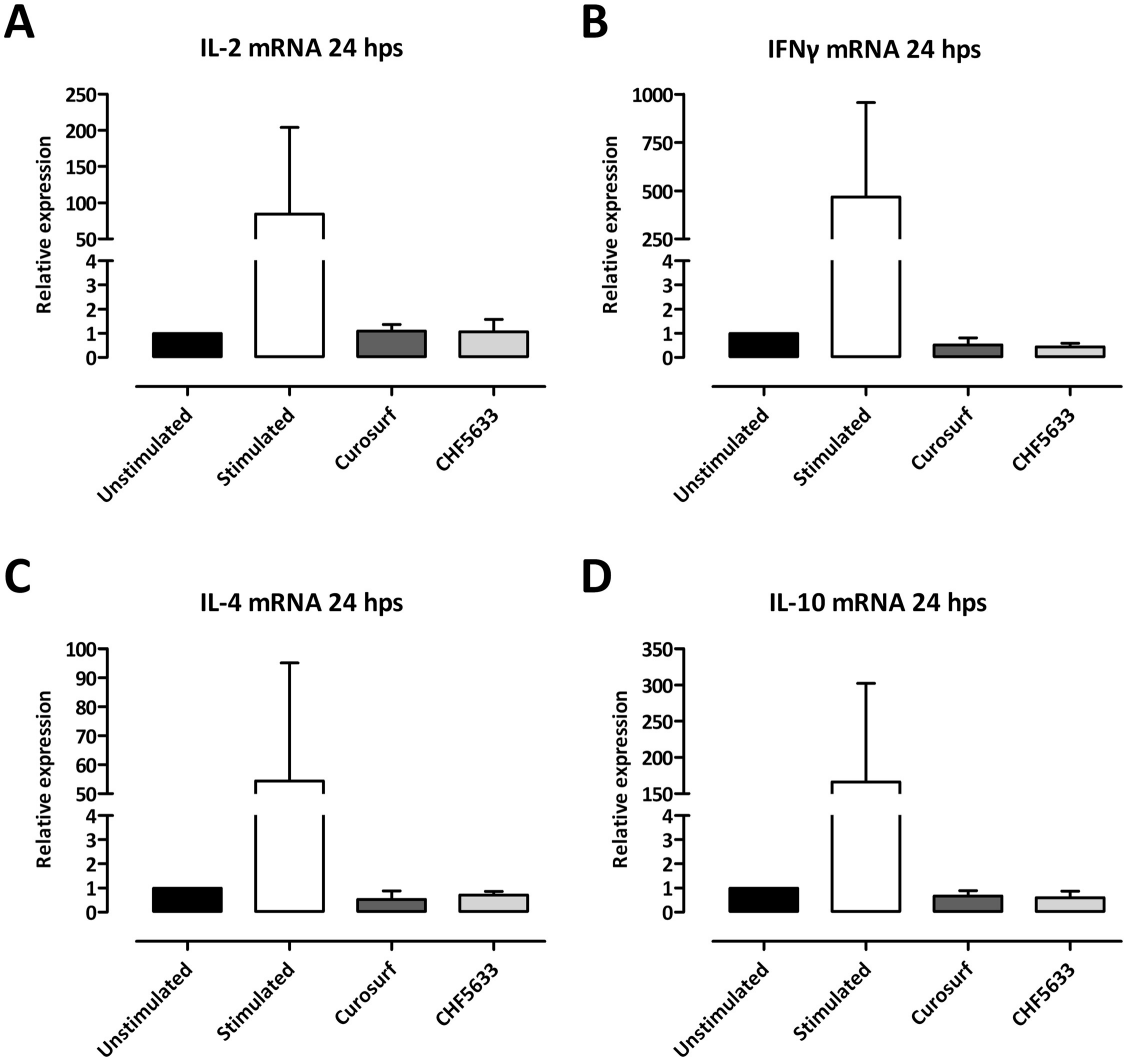
Influence of surfactant preparations on cytokine mRNA expression of resting CD4^+^ lymphocytes. Unactivated CD4^+^ lymphocytes were either left untreated or incubated with 100 μg/mL Curosurf^®^ or CHF5633 and 24 h later total RNA was isolated and mRNAs of IL-2 (**A**), IFNγ (**B**), IL-4 (**C**), and IL-10 (**D**) were quantified by qPCR. Means + SD of n = 3 independent experiments are shown. Hps, hours post stimulation.

CHF5633 as well as Curosurf^®^ did not affect mRNA expression of IL-2, IFNγ, IL-4, or IL-10 of resting CD4^+^ T cells in comparison to untreated controls (Fig 3). Different combinations of CHF5633’s single components also showed no significant modulatory effects (S3 Fig).

In activated cells, cytokine mRNA levels were significantly induced after 24 h (unpaired *t*-test) in comparison to resting cells for IL-2 (62 ± 73-fold, *p* < 0.05), IFNγ (480 ± 410-fold, *p* < 0.01), IL-4 (130 ± 55-fold, *p* < 0.0001), IL-10 (290 ± 240-fold, *p* < 0.01), and IL-17A (260 ± 240-fold, *p* < 0.01). Only IL-22 mRNA induction was found to be highly inhomogeneous, ranging from 5.1-fold to 5200-fold in comparison to resting cells.

**Fig 4:**
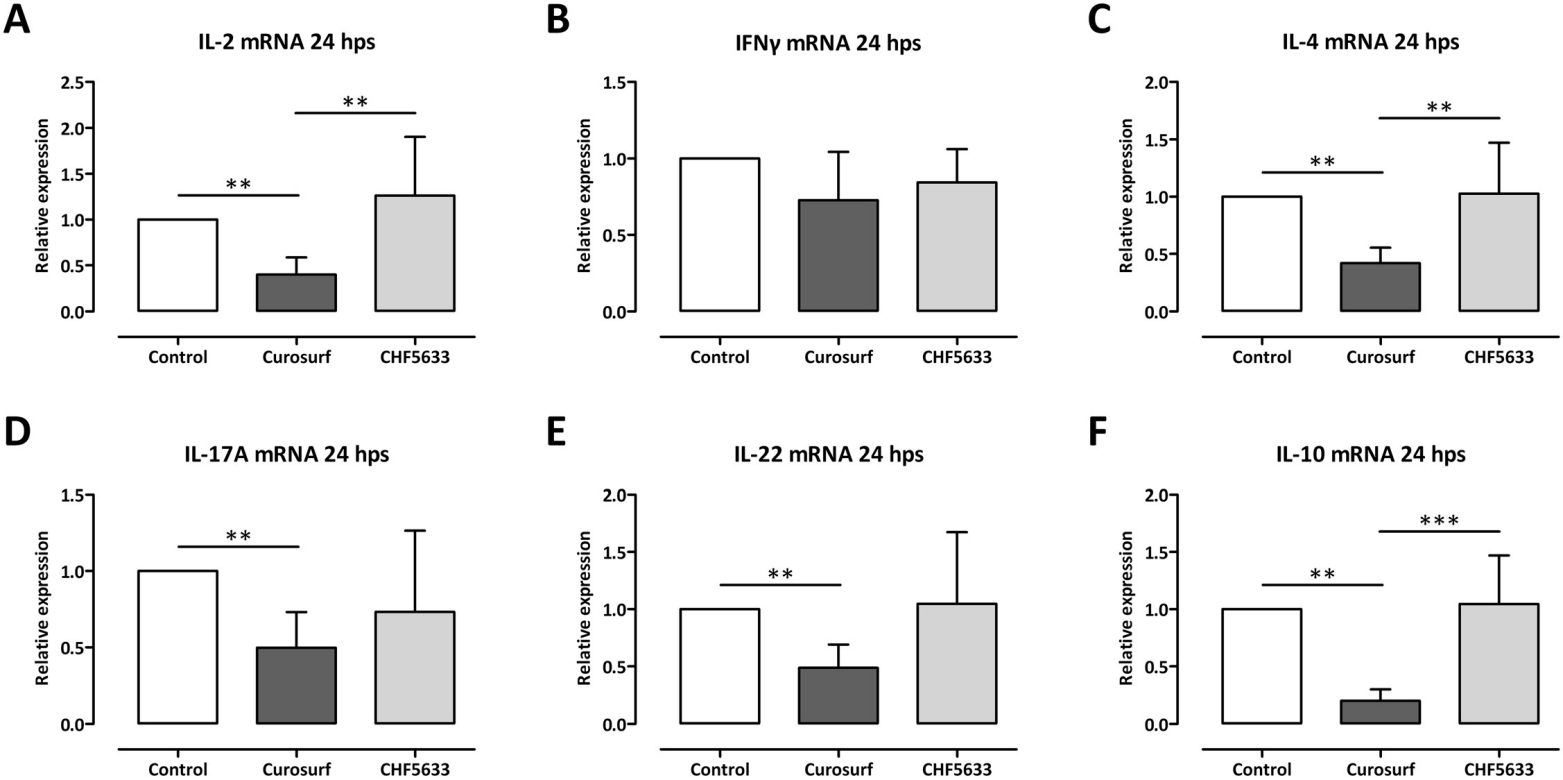
Influence of surfactant preparations on cytokine mRNA expression of activated CD4^+^ lymphocytes. CD3/CD28 activated CD4^+^ lymphocytes were either left untreated or incubated with 100 μg/mL Curosurf^®^ or CHF5633 and 24 h later total RNA was isolated and mRNAs of IL-2 (**A**), IFNγ (**B**), IL-4 (**C**), IL-17A (**D**), IL-22 (**E**), and IL-10 (**F**) were quantified by qPCR. Means + SD of n = 6 independent experiments for IL-22 mRNA or n = 8 independent experiments for all other cytokine mRNAs are shown. Hps, hours post stimulation; ** *p* < 0.01; *** *p* < 0.001.

No significant differences between untreated activated cells and CHF5633-exposed activated cells were found for all investigated cytokine mRNAs (Fig 4). In contrast, a significant reduction of cytokine mRNAs in Curosurf^®^-treated cells in comparison to untreated controls and CHF5633-treated cells was found for IL-2 (0.4 ± 0.19-fold, *p* < 0.01 and 0.32 ± 0.15-fold, *p* < 0.01, respectively; Fig 4A), IL-4 (0.42 ± 0.14-fold, *p* < 0.01 and 0.41 ± 0.13-fold, *p* < 0.01, respectively; Fig 4C), and IL-10 (0.2 ± 0.1-fold, *p* < 0.01 and 0.19 ± 0.1-fold, *p* < 0.001, respectively; Fig 4F). Cytokine mRNA reduction in Curosurf^®^-treated cells only in comparison to untreated controls was found for IL-17A (0.49 ± 0.23-fold, *p* < 0.01; Fig 4D) and IL-22 (0.49 ± 0.21-fold, *p* < 0.01; Fig 4E). No significant differences of either activated and Curosurf^®^- or CHF5633-treated CD4^+^ lymphocytes in comparison to activated and untreated cells were found for IFNγ (Fig 4B).

### Cytokine expression in resting and activated CD4^+^ lymphocytes

We measured levels of intracellularly accumulated IL-2, IFNγ, IL-4, and IL-10 in comparison to untreated cells after a 40 h incubation. At this timepoint, all of these cytokines were simultaneously detectable if the cells were activated by anti CD3/CD28 antibodies. In comparison to resting cells, the percental amount of cytokine-positive cells was very inhomogeneous for each cytokine, being 7.4 ± 1.1 for IL-2, 50 ± 64 for IFNγ, 11 ± 14 for IL-4, and 190 ± 170 for IL-10 after 40 h.

**Fig 5:**
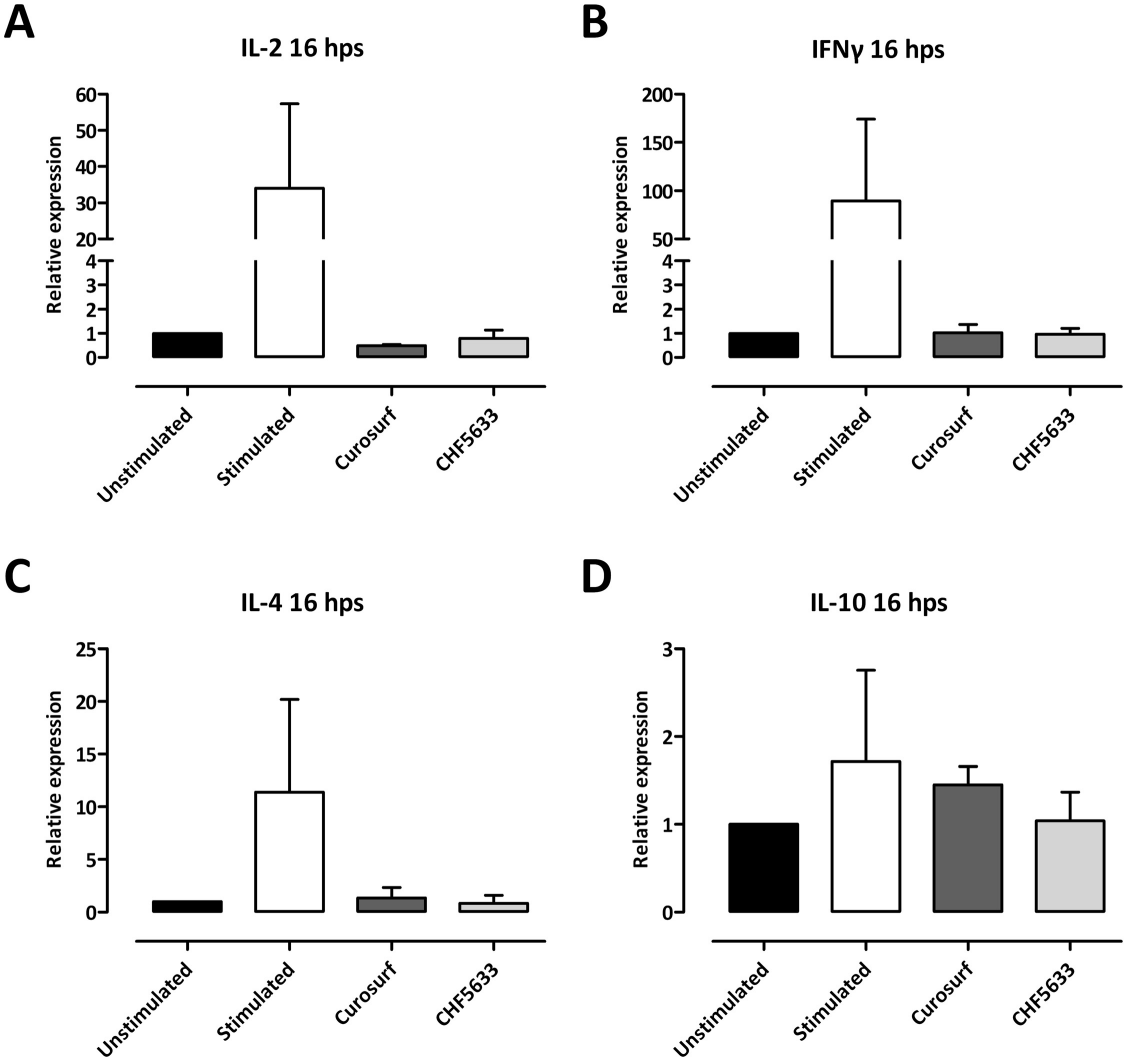
Influence of surfactant preparations on cytokine expression of resting CD4^+^ lymphocytes. Unactivated CD4^+^ lymphocytes were either left untreated or incubated with 100 μg/mL Curosurf^®^ or CHF5633 and 16 h later intracellular enriched cytokines were measured by flow cytometry. Vital CD4^+^ lymphocytes were gated as shown in Fig 6A and analyzed for IL-2 (**A**), IFNγ (**B**), IL-4 (**C**), and IL-10 (**D**) expression. Means + SD of n = 3 independent experiments are shown. Hps, hours post stimulation.

As shown for mRNA expression, neither CHF5633 (Fig 5) nor different combinations of its single components (S4 Fig) had any significant modulatory effects on intracellular expression of IL-2, IFNγ, IL-4, or IL-10 in resting CD4^+^ T cells without activation in comparison to untreated controls, respectively. The same was again also true for exposure to Curosurf^®^ (Fig 5).

**Fig 6:**
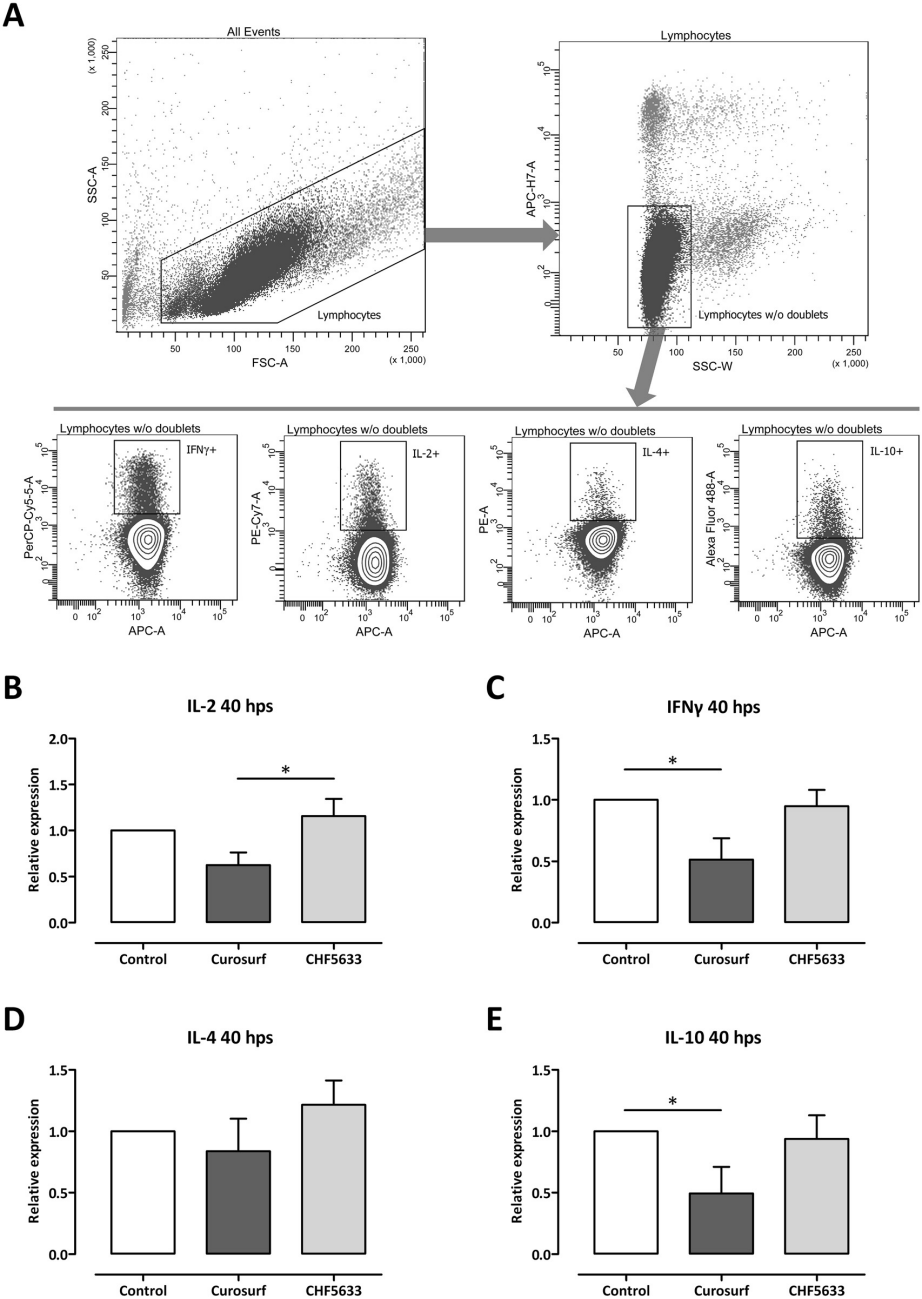
Influence of surfactant preparations on cytokine expression of activated CD4^+^ lymphocytes. CD3/CD28 activated CD4^+^ lymphocytes were either left untreated or incubated with 100 μg/mL Curosurf^®^ or CHF5633 and 40h later intracellular enriched cytokines were measured by flow cytometry. Vital CD4^+^ lymphocytes were gated as shown in **A** and analyzed for IL-2 (**B**), IFNγ (**C**), IL-4 (**D**), and IL-10 (**E**) expression. Means + SD of n = 4 independent experiments are shown. Hps, hours post stimulation; * *p* < 0.05.

After activation via CD3/CD28, no significant differences between untreated cells and CHF5633-treated cells were found for all intracellularly accumulated cytokines (Fig 6). A significant reduction of cytokine production of Curosurf^®^-treated cells in comparison to untreated controls was found for IFNγ (0.51 ± 0.17-fold, *p* < 0.05; Fig 6C) and IL-10 (0.49 ± 0.22-fold, *p* < 0.05; Fig 6E), while a significant reduction of cytokine production of Curosurf^®^-treated cells in comparison to CHF5633-treated cells was found for IL-2 (0.54 ± 0.12-fold, *p* < 0.05; Fig 6B). The gating strategy for these experiments is shown in Fig 6A.

### Influence of single components of CHF5633 on cytokines and cytokine mRNA expression in activated CD4^+^ lymphocytes

For CHF5633’s synthetic sub-components we found a significant increase of cytokine mRNA for IL-4 in POPG-treated activated cells in comparison to POPG+DPPC-treated activated cells (1.92 ± 0.65-fold, *p* < 0.05; S5C Fig), whereas a significant reduction was found for IL-17A in POPG-treated activated cells in comparison to untreated activated cells (0.36 ± 0.23-fold, *p* < 0.01; S5E Fig). The mRNA expression of all other cytokines was not significantly different for all other tested combinations of CHF5633 components (S5 Fig). Except for a significant reduction of intracellularly accumulated IFNγ in POPG-treated activated CD4^+^ lymphocytes in comparison to untreated activated controls (0.49 ± 0.16-fold, *p* < 0.01; S6B Fig) this was also the case for every other tested combination of the CHF5633 components for all tested cytokines (IL-2, IFNγ, IL-4, and IL-10; S6 Fig).

## Discussion

To our knowledge, no study has ever focused on a potential impact of surfactant preparations on purified human CD4^+^ cells until now, but only analyzed effects on PBMCs [4]. Here, we expand prior work about immunomodulatory properties of surfactant preparations by investigating potential effects of the new generation reconstituted synthetic surfactant CHF5633 and its components on cell viability, proliferation, and pro- and anti-inflammatory cytokine responses in human CD4^+^ lymphocytes, representing an important part of the adaptive immunity in the lung.

Our data demonstrated that CHF5633 is not cytotoxic to CD4^+^ T cells and has no impact on their proliferative ability under the given conditions. In PBMCs, an inhibitory capacity of various animal derived surfactants on cell proliferation *in vitro* was described [4]. In contrast to work identifying phosphatidylglycerol as the main anti-proliferative agent of animal derived surfactant [23, 26], we could not detect a reduction of the proliferative response for the defined surfactant concentrations, including POPG. Because mainly PBMCs were used in those studies, proliferation of CD4^+^ T cells may be indirectly influenced by cell types additionally present and producing effector cytokines as well [47]. In addition, the type of stimulation may be very important for the outcome and differs for our setting, since we used anti CD3/CD28 antibodies instead of phytohaemagglutinin or concanavalin A. Furthermore, simultaneous incubation, like in our context, and pre- or postincubation with surfactant in combination with other stimuli can lead to distinct responses, as already described for splenocytes and monocytes [28]. However, expansion is of great importance for a proper implementation of CD4^+^ effector function in the lung [48], and an unaffected proliferation of residing CD4^+^ lymphocytes after treatment with exogenous surfactant might be beneficial in terms of controlling lung inflammation.

To facilitate their regulating task, CD4^+^ lymphocytes secrete cytokines which modulate fate and function of other cells [47]. In the absence of inflammatory stimuli, no induction of pro-inflammatory cytokine response after CHF5633 exposure could be observed in resting CD4^+^ lymphocytes in comparison to non-exposed controls on the transcriptional and the translational level, indicating that CHF5633 cannot induce inflammation *per se*. This result is consistent with a report describing lower neutrophil counts in alveolar spaces of preterm lambs, indicating lower inflammatory attraction of CHF5633 in comparison to Curosurf^®^ [11].

Considering that lung inflammation may be a constant problem in preterm infants due to infection, chorioamnionitis, or ventilation [49], we simulated a simultaneous inflammatory trigger *in vitro* by activating CD4^+^ T cells using CD3/CD28 antibodies during exposure to surfactant. CHF5633 did not enhance pro-inflammatory cytokines IL-2 and IFNγ or influence anti-inflammatory cytokines IL-4 and IL-10 on the transcriptional and the translational level. In addition, the expression of the Th17-specific cytokine mRNAs of IL-17A and IL-22 were also not modulated. In good agreement with data on PBMCs [34], the lacking influence of CHF5633 on IL-2 might also explain the unaltered CD4^+^ lymphocyte proliferation induced by CD3/CD28. Since it has been shown that the ability of IL-2 to increase expression of IL-4 and IFNγ is higher than those of IL-15 [50], the latter has not been included in our investigation.

Likewise, different combinations of CHF5633’s single components also had no effect on cytokine responses on the transcriptional as well as the translational level. Only pure POPG was able to significantly modify the CD3/CD28 induced expression of IL-4 and IL17A on the transcriptional and of IFNγ on the translational level. This is indicating that the modulatory ability of POPG, which has already been shown for rat and human alveolar macrophages [51], might also be true for CD4^+^ lymphocytes although to a lesser extent.

However, in view of the fact that CHF5633 did not induce pro-inflammatory cytokines in resting as well as activated CD4^+^ lymphocytes in our *in vitro* setting, this might further support its application in surfactant replacement therapy, particularly because batch-to-batch variations are no longer relevant [52].

In order to examine also the influence of a natural surfactant, we used Curosurf^®^ for exposure to highly purified CD4^+^ lymphocytes, which has in this way also never been addressed before. In accordance with previous data on PBMCs and natural surfactants [4], a mild trend of less vital and proliferated cells as well as slightly higher amount of completely unproliferated CD4^+^ lymphocytes could be detected after exposure to Curosurf^®^ in comparison to controls. Likewise in line with described effects for PBMCs by our and other groups [13, 19, 20], we could show that Curosurf^®^ is able to significantly reduce cytokine expression on the transcriptional and the translational level. We detected significantly lower mRNA levels of the pro-inflammatory IL-2, the anti-inflammatory IL-4 and IL-10, and the Th17-specific IL17A and IL22. Analogous to data for rat splenocytes exposed to porcine surfactant [53], we found significantly reduced protein levels of IFNγ and IL-10 in Curosurf^®^-exposed CD4^+^ cells in comparison to surfactant-unexposed cells. Because anti- as well as pro-inflammatory cytokines seem to be comparably affected, this might well indicate an overall reductive ability of natural surfactants in the case of CD4^+^ T cells. However, the final influence of Curosurf^®^ on the pro- and anti-inflammatory balance *in vivo* can not be predicted because only a single cell type was investigated *in vitro*. In contrast, existing *in vivo* data points to an overall reduction of inflammation by Curosurf^®^ in a rat model of early-stage acute RDS [54]. Since DPPC and POPG are components in both the animal-derived Curosurf^®^ and CHF5633, it is most likely that the observed influences of the natural preparation in comparison to the fully synthetic and reconstituted CHF5633 are therefore attributable to an involvement of other phospholipid species not present in CHF5633 [55].

The tendency towards enhanced anti-inflammatory cytokine expression in direct comparison to Curosurf^®^ is in accordance to data for CHF5633 and CD14^+^ monocytes by our group [56] and work describing higher IL-10 expression in monocytes after incubation with synthetic surfactant [21]. Thus, the general, non-modulatory property of CHF5633 may also be beneficial in view of anti-inflammatory effects.

However, there are some limitations of this study worth mentioning. One major limitation may be the usage of adult, CD3/CD28-activated CD4^+^ lymphocytes as a readout model for cytokine response, although surfactant replacement therapy is implemented in preterm neonates comprising multiple inflammatory stimuli. For this reason, future studies will address the same issue for CD4^+^ lymphocytes isolated from cord blood using different triggers of activation. Furthermore to map changes simultaneously, only one time frame was chosen to reflect modifications in cytokine expression on the transcriptional and the translational level, respectively, although individual kinetics exist for each cytokine. Since it has been shown that lung resident lymphocytes may act differently in response to infection [57, 58], it can be assumed that this may also be the case for stimuli such as synthetic surfactant preparations. Therefore, the findings of the present study may not completely resemble situations in premature infants. In addition to already existing studies investigating biophysical effectiveness, further work should also examine potential immunomodulatory abilities of new generation surfactants *in vivo*.

### Conclusion

CHF5633 did not exhibit any cytotoxicity or pro-inflammatory activity in non-activated and activated human CD4^+^ lymphocytes under the given conditions. With regard to anti-inflammatory cytokines, CHF5633 might also lack an overall reductive ability in comparison to animal-derived surfactants, possibly leaving pro- and anti-inflammatory cytokine response in balance. Hence, our *in vitro* data may point to a supportive role of CHF5633 in improving lung function without inducing or exacerbating lung inflammation.

## Supporting Information

S1 Fig Influence of single components of CHF5633 on vitality of activated CD4^+^ lymphocytes. CD3/CD28 activated CD4^+^ lymphocytes were either left untreated or incubated with different combinations of CHF5633’s components for 48 h (**A**) or 72 h (**B**), followed by measurement of cell viability by using MTT. Means + SD of n = 5 independent experiments are shown. Hps, hours post stimulation.
(TIF)

S2 Fig Influence of single components of CHF5633 on proliferation ability of activated CD4^+^ lymphocytes. CD3/CD28 activated CD4^+^ lymphocytes were either left untreated or incubated with different combinations of CHF5633’s components and 72 h later proliferation was determined by means of CFSE. Vital CFSE-positive CD4^+^ lymphocytes were gated as shown in Fig 2A. No significant differences between total proliferated cells (**A**) or ratios of different amounts of divisions (**B**) were found after treatment with different combinations of CHF5633’s components in comparison to untreated cells. Means + SD of n = 6 independent experiments are shown. Hps, hours post stimulation.
(TIF)

S3 Fig Influence of single components of CHF5633 on cytokine mRNA expression of resting CD4^+^ lymphocytes. Unactivated CD4^+^ lymphocytes were either left untreated or incubated with different combinations of CHF5633’s components as indicated and 24 h later total RNA was isolated and mRNAs of IL-2 (**A**), IFNγ (**B**), IL-4 (**C**), and IL-10 (**D**) were quantified by qPCR. Means +SD of n = 3 independent experiments are shown. Hps, hours post stimulation.
(TIF)

S4 Fig Influence of single components of CHF5633 on cytokine expression of resting CD4^+^ lymphocytes. Unactivated CD4^+^ lymphocytes were either left untreated or incubated with different combinations of CHF5633’s components as indicated and 16 h later intracellular enriched cytokines were measured by flow cytometry. Vital CD4^+^ lymphocytes were gated as shown in Fig 6A and analyzed for IL-2 (**A**), IFNγ (**B**), IL-4 (**C**), and IL-10 (**D**) expression. Means +SD of n = 3 independent experiments are shown. Hps, hours post stimulation.
(TIF)

S5 Fig Influence of single components of CHF5633 on cytokine mRNA expression of activated CD4^+^ lymphocytes. CD3/CD28 activated CD4^+^ lymphocytes were either left untreated or incubated with CHF5633 or different combinations of its components as indicated and 24 h later total RNA was isolated and mRNAs of IL-2 (**A**), IFNγ (**B**), IL-4 (**C**), IL-10 (**D**), IL-17A (**E**), and IL-22 (**F**) were quantified by qPCR. Means +SD of n = 6 independent experiments for IL-22 mRNA or n = 8 independent experiments for all other cytokine mRNAs are shown. Hps, hours post stimulation; * *p* < 0.05; ** *p* < 0.01.
(TIF)

S6 Fig Influence of single components of CHF5633 on cytokine expression of activated CD4^+^ lymphocytes. CD3/CD28 activated CD4^+^ lymphocytes were either left untreated or incubated with CHF5633 or different combinations of its components as indicated and 40 h later intracellular enriched cytokines were measured by flow cytometry. Vital CD4^+^ lymphocytes were gated as shown in Fig 6A and analyzed for IL-2 (**A**), IFNγ (**B**), IL-4 (**C**), and IL-10 (**D**) expression. Means + SD of n = 4 independent experiments are shown. Hps, hours post stimulation; ** *p* < 0.01.
(TIF)
