# Supplementary figures and images for: Impact of the New Generation Reconstituted Surfactant CHF5633 on Human CD4+ Lymphocytes
Source: PLoS One. 2016 Apr 14;11(4):e0153578. doi: 10.1371/journal.pone.0153578 (PMC4831819; doi:10.1371/journal.pone.0153578)

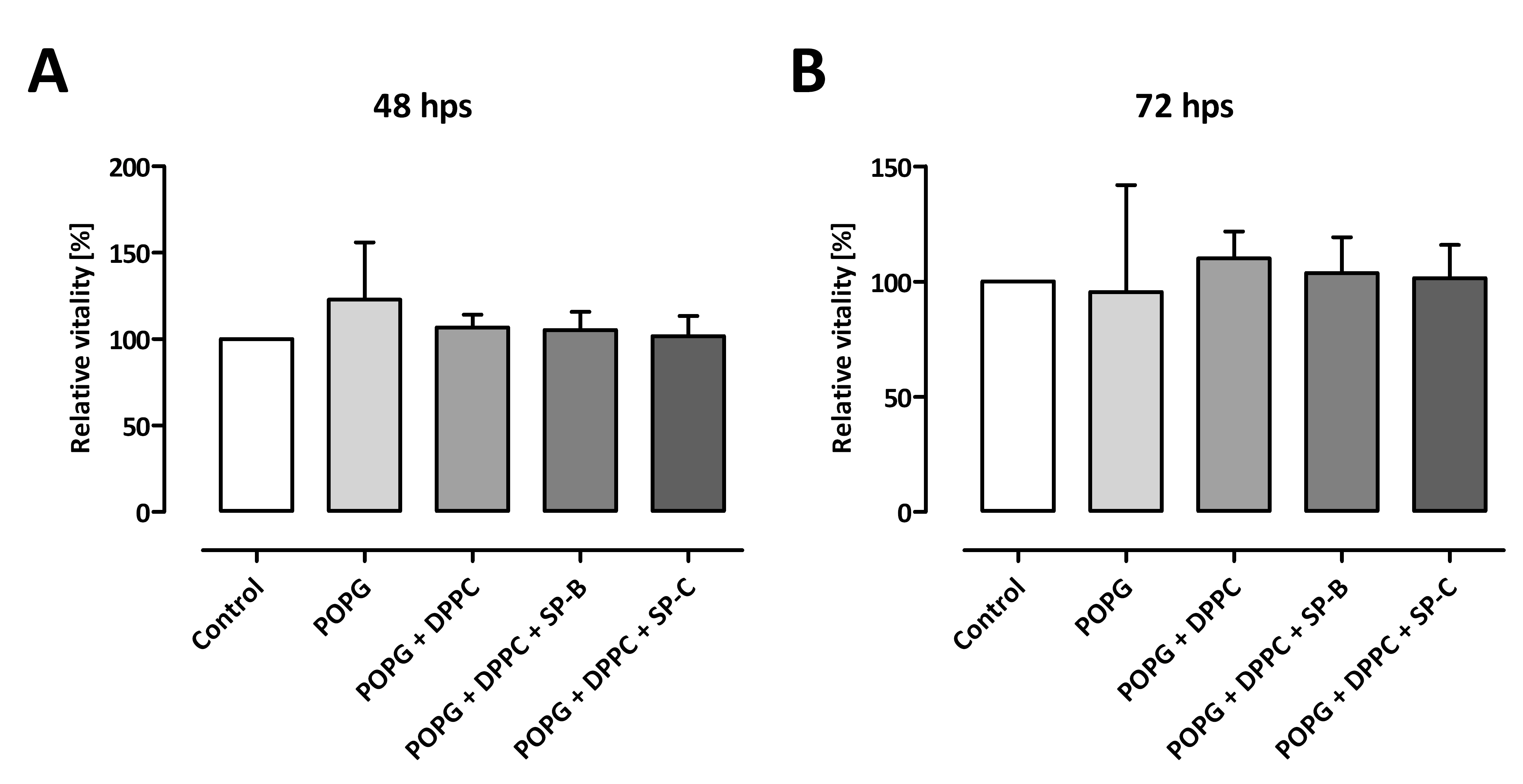

Supplement: S1 Fig — CD3/CD28 activated CD4+ lymphocytes were either left untreated or incubated with different combinations of CHF5633’s components for 48 h (A) or 72 h (B), followed by measurement of cell viability by using MTT. Means + SD of n = 5 independent experiments are shown. Hps, hours post stimulation. (TIF) [file pone.0153578.s001.tif]

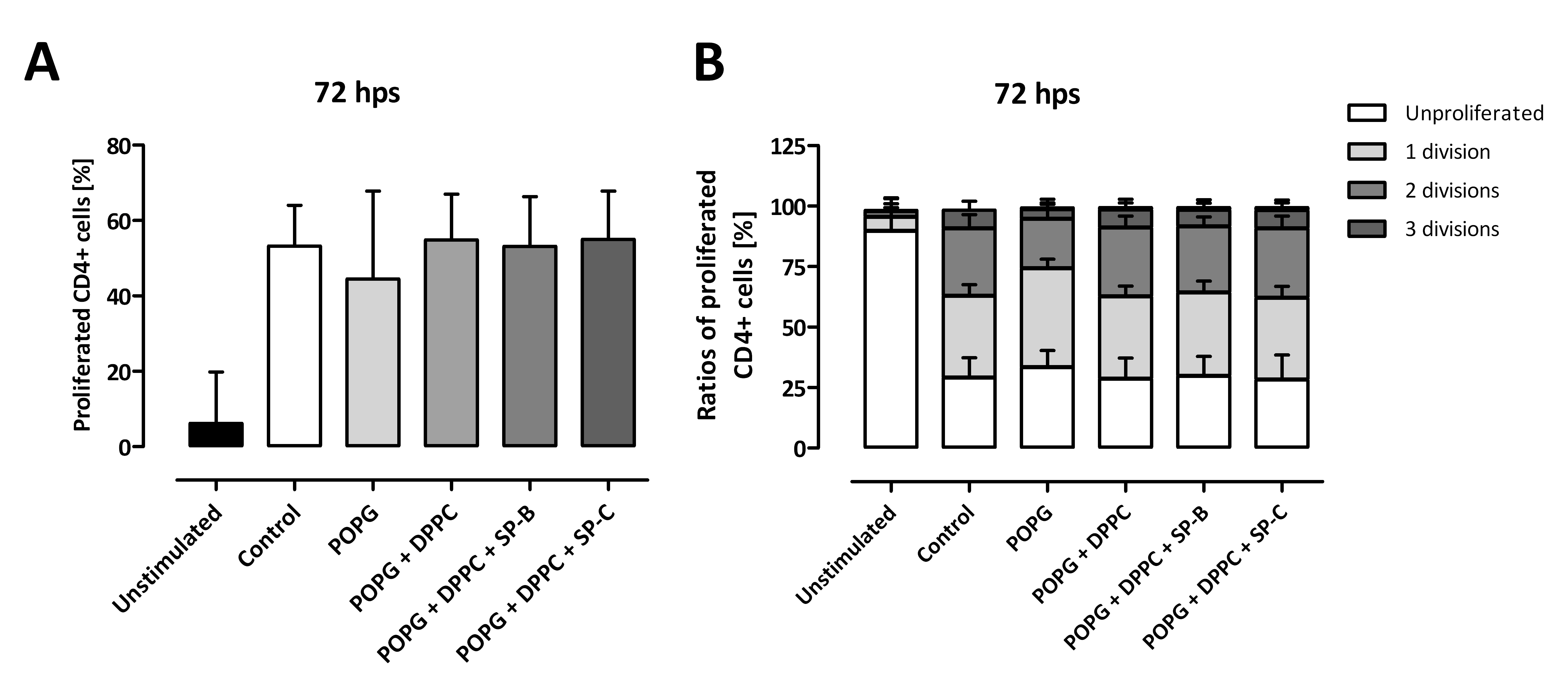

Supplement: S2 Fig — CD3/CD28 activated CD4+ lymphocytes were either left untreated or incubated with different combinations of CHF5633’s components and 72 h later proliferation was determined by means of CFSE. Vital CFSE-positive CD4+ lymphocytes were gated as shown in Fig 2A. No significant differences between total proliferated cells (A) or ratios of different amounts of divisions (B) were found after treatment with different combinations of CHF5633’s components in comparison to untreated cells. Means + SD of n = 6 independent experiments are shown. Hps, hours post stimulation. (TIF) [file pone.0153578.s002.tif]

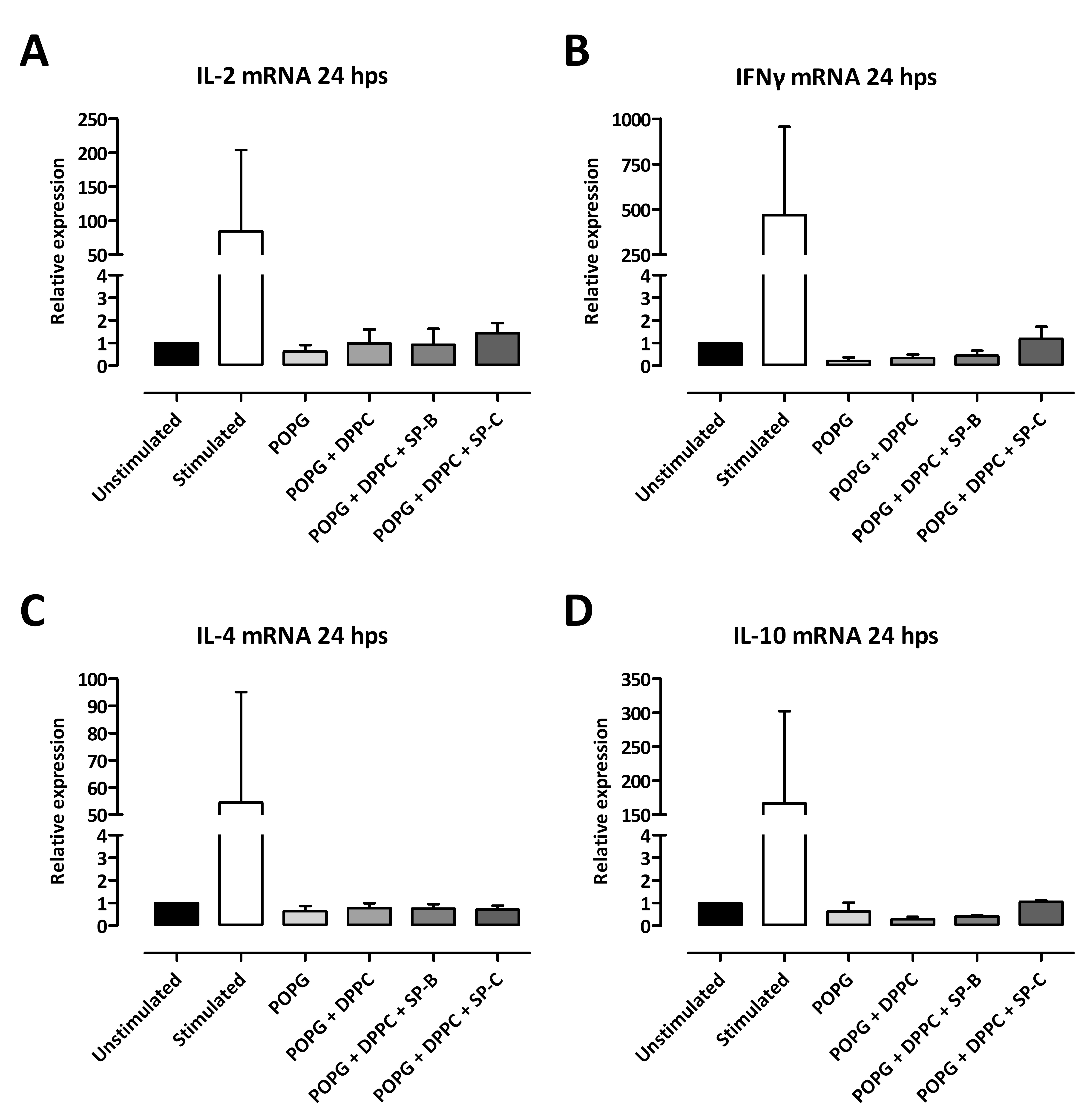

Supplement: S3 Fig — Unactivated CD4+ lymphocytes were either left untreated or incubated with different combinations of CHF5633’s components as indicated and 24 h later total RNA was isolated and mRNAs of IL-2 (A), IFNγ (B), IL-4 (C), and IL-10 (D) were quantified by qPCR. Means +SD of n = 3 independent experiments are shown. Hps, hours post stimulation. (TIF) [file pone.0153578.s003.tif]

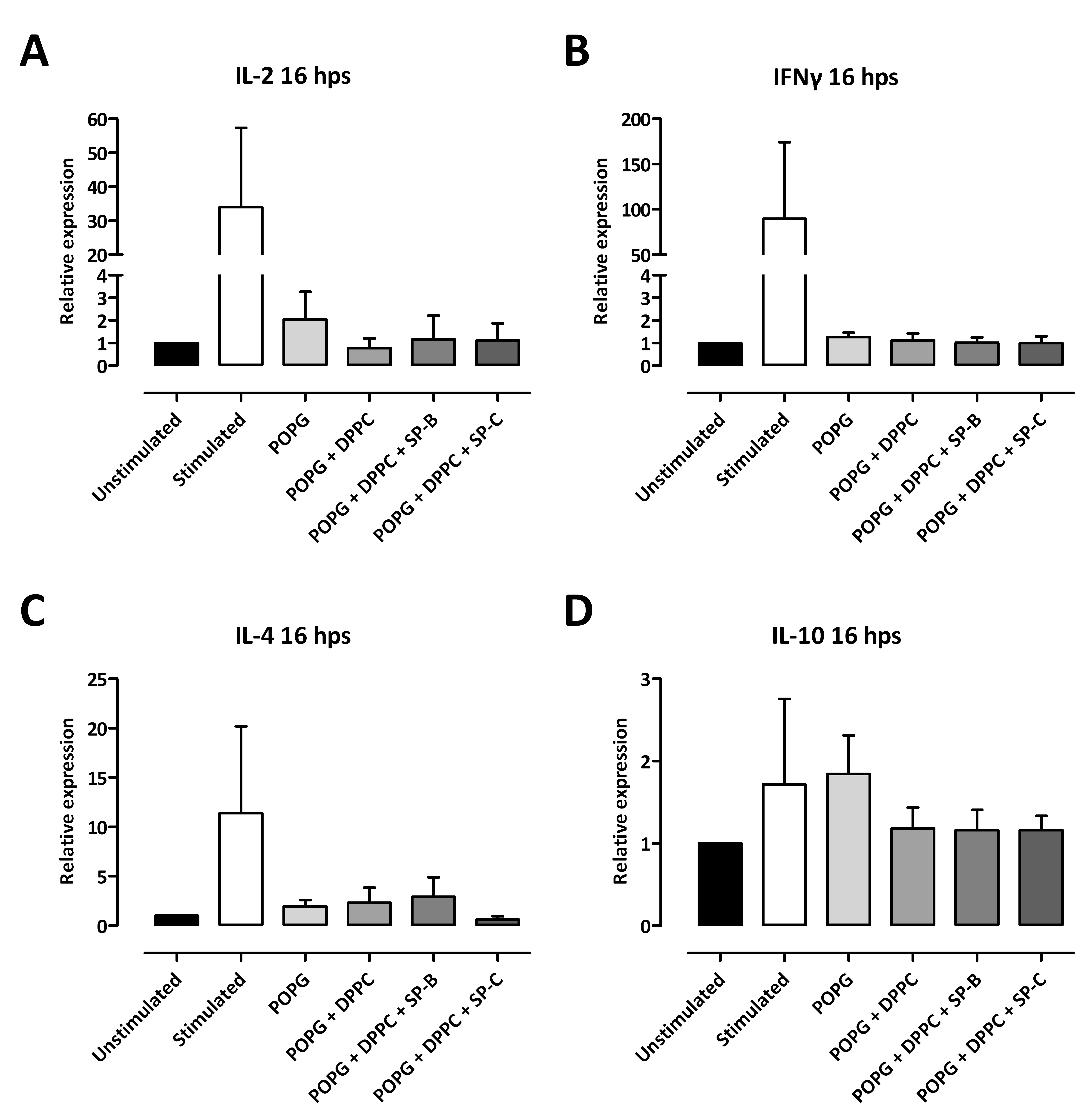

Supplement: S4 Fig — Unactivated CD4+ lymphocytes were either left untreated or incubated with different combinations of CHF5633’s components as indicated and 16 h later intracellular enriched cytokines were measured by flow cytometry. Vital CD4+ lymphocytes were gated as shown in Fig 6A and analyzed for IL-2 (A), IFNγ (B), IL-4 (C), and IL-10 (D) expression. Means +SD of n = 3 independent experiments are shown. Hps, hours post stimulation. (TIF) [file pone.0153578.s004.tif]

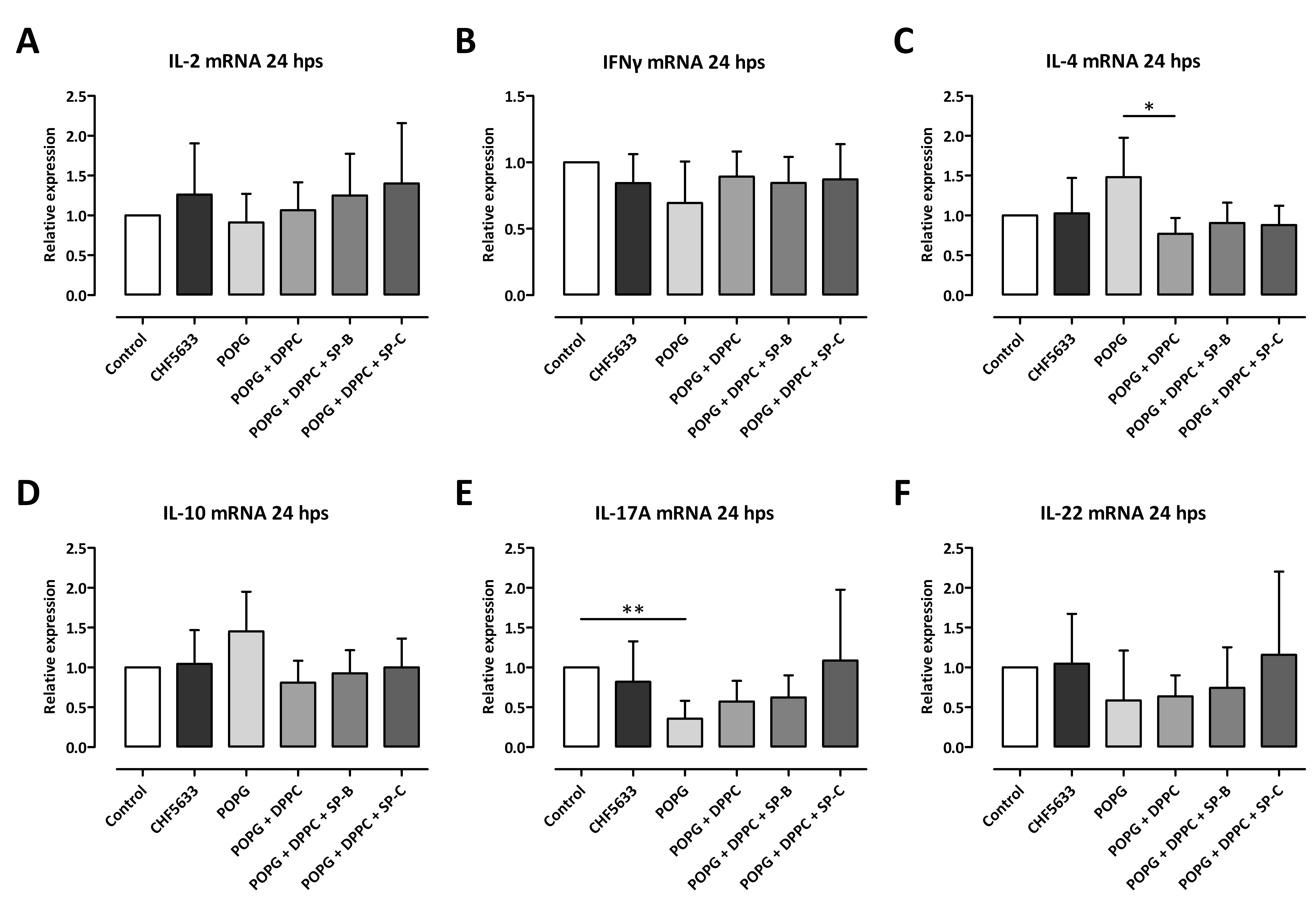

Supplement: S5 Fig — CD3/CD28 activated CD4+ lymphocytes were either left untreated or incubated with CHF5633 or different combinations of its components as indicated and 24 h later total RNA was isolated and mRNAs of IL-2 (A), IFNγ (B), IL-4 (C), IL-10 (D), IL-17A (E), and IL-22 (F) were quantified by qPCR. Means +SD of n = 6 independent experiments for IL-22 mRNA or n = 8 independent experiments for all other cytokine mRNAs are shown. Hps, hours post stimulation; * p < 0.05; ** p < 0.01. (TIF) [file pone.0153578.s005.tif]

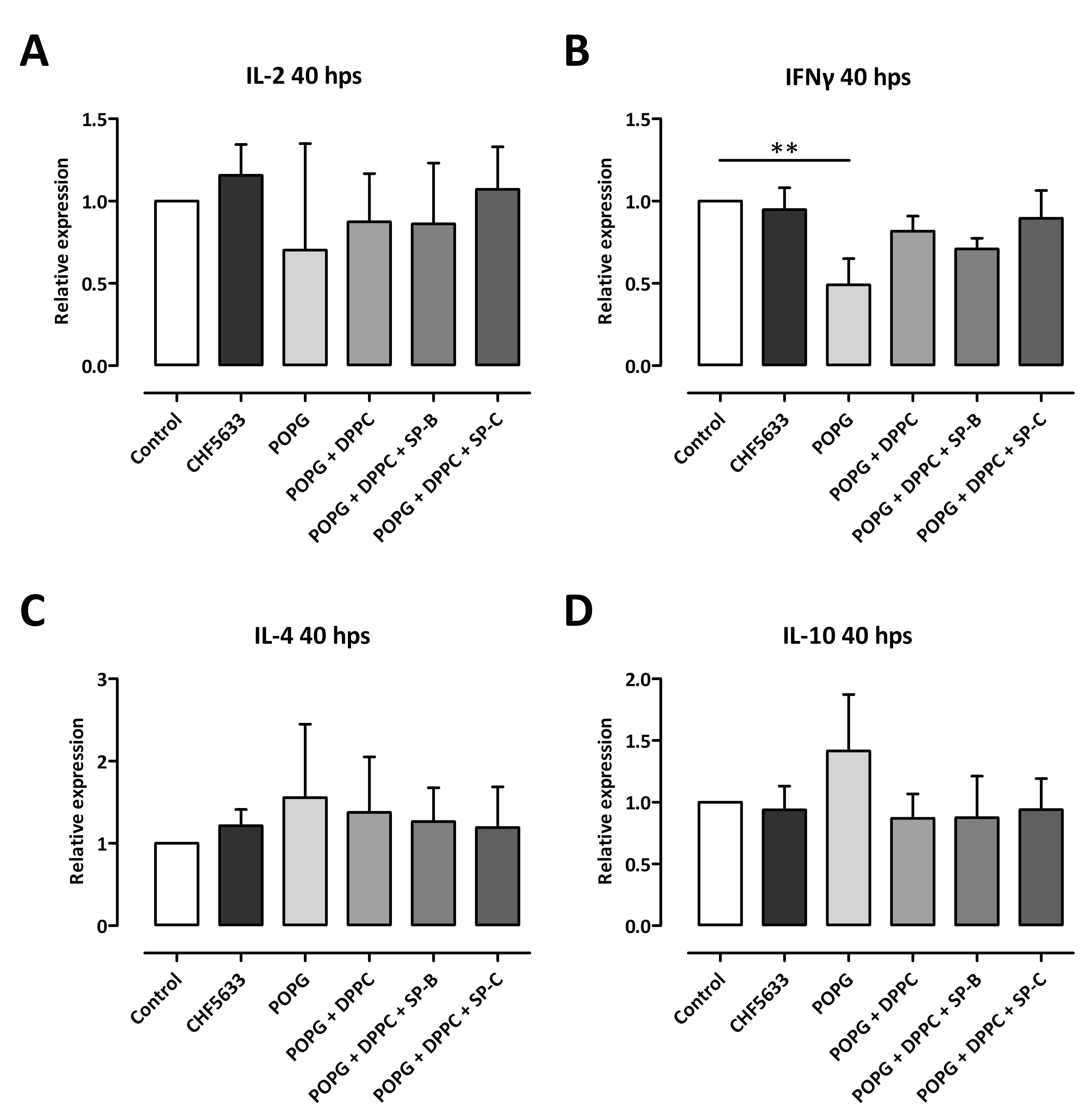

Supplement: S6 Fig — CD3/CD28 activated CD4+ lymphocytes were either left untreated or incubated with CHF5633 or different combinations of its components as indicated and 40 h later intracellular enriched cytokines were measured by flow cytometry. Vital CD4+ lymphocytes were gated as shown in Fig 6A and analyzed for IL-2 (A), IFNγ (B), IL-4 (C), and IL-10 (D) expression. Means + SD of n = 4 independent experiments are shown. Hps, hours post stimulation; ** p < 0.01. (TIF) [file pone.0153578.s006.tif]
